# A review of optimization strategies for deep and machine learning in diabetic macular edema

**DOI:** 10.3389/frai.2026.1684752

**Published:** 2026-02-13

**Authors:** A. M. Mutawa, Khalid Sabti, Bibin Shalini Sundaram Thankaleela, Seemant Raizada

**Affiliations:** 1 Department of Computer Engineering, College of Engineering and Petroleum, Kuwait University, Sabah Al Salem University City, Shadadiya, Kuwait; 2 Kuwait Specialized Eye Center, Kuwait City, Kuwait

**Keywords:** deep learning, DME, machine learning, optimization, soft computing, sunburst diagram

## Abstract

Diabetic macular edema (DME) is a primary contributor to visual impairment in diabetic patients, necessitating precise and prompt analysis for optimal treatment. Recent breakthroughs in deep learning (DL) and machine learning (ML) have yielded promising outcomes in ophthalmic image analysis. However, researchers often overlook the significance of optimization algorithms in enhancing the efficacy of their models for DME-related tasks. This review aims to consolidate, seek, discover, assess, and integrate existing work on the application of DL and ML, with emphasis on the integration and impact of optimization algorithms in enhancing their efficacy, robustness, and performance for DME in the fields of computer science and engineering. The population, intervention, comparison, and outcome framework was employed in this study to facilitate a clear and comprehensive analysis. The procedural superiority of the included investigations was evaluated using the Joanna Briggs Institute Critical Appraisal Tools for assessing methodological quality. The Auto-Metric Graph Neural Network achieved the greatest accuracy of 99.57% for combined diabetic retinopathy-DME grading, illustrating the higher efficacy of hybrid architectures augmented by meta-heuristic optimizers, such as Jaya and ant colony optimization. Successful deployment, however, depends on overcoming hurdles, such as the low mean average precision in lesion identification (0.1540) in YOLO-based models in the test set performance, and improved clinical interpretability to foster clinician trust. A Sankey diagram visually analyzes the flow of quantities between different entities of the survey.

**Systematic review registration:**

B. (2025, November 2). A Review of Optimization Strategies for Deep and Machine Learning in DME. Retrieved from osf.io/qsh4j.

## 1 Introduction

Regular eye screenings are crucial for the early detection and treatment of diabetic eye diseases, such as diabetic macular edema (DME) and diabetic retinopathy (DR), to prevent vision loss. If neglected, diabetic eye problems can cause irreparable retinal damage, leading to vision impairment or total blindness (Gulati et al., 2024).

DME is a serious condition triggered by retinal vein obstruction, DR, chronic uveitis, and eye injury. It is characterized by retinal thickening and fluid accumulation in the retinal layers (Feng et al., 2023). DME affects the fovea and results from capillary leakage and the buildup of retinal fluid (Toto et al., 2024). When compromised retinal blood vessels become engorged and begin exuding protein and fluid onto the retinal surface (Ajaz et al., 2021). The illness is complex, arising from multiple pathophysiological pathways associated with angiogenesis and altered permeability of the blood-retinal barrier (Blanot et al., 2024).

Advancements in medical imaging technology have revolutionized the diagnosis and management of retinal problems. Optical coherence tomography (OCT) is a high-resolution imaging procedure used for the initial recognition of ocular pathologies, such as DME, by projecting cross-sectional images from the human iris. It can also be used in computer-aided diagnosis systems (Tajmirriahi et al., 2022). OCT employs low-coherence light to generate cross-sectional images of ocular biological tissue with micro-level resolution. Due to its non-invasive imaging collection, OCT is widely preferred for evaluating retinal diseases (Ge et al., 2022). Color fundus photography (CFP) is a clinical tool for assessing ME/DME advancement, providing a flat, wide-angle view of the retina and crucial insights into its health while detecting signs of DME, such as hard exudates (HE) and retinal thickening. Fundus imaging can be mydriatic or non-mydriatic, the former providing improved, high-quality images. However, its inability to compute retinal stiffness limits its efficacy in analysis and management. Fluorescein angiography (FA) is a technique that uses a fluorescent dye to visualize blood flow in the optic nerve, enabling the detection of choroidal and retinal circulations through various filters. Macular edema can be identified through the patterns of leakage and macular perfusion observed on FA. Optical coherence tomography angiography (OCTA) is a non-invasive imaging system that provides a quantitative assessment of retinal vasculature and offers three-dimensional representations of the macula. It captures reflections of laser light from the surfaces of moving blood cells to visualize retinal arteries (Ajaz et al., 2021).

AI technologies have leveraged these advancements to automate the study of biomarkers, guiding disease diagnosis and therapy decisions. The retinal fluid has emerged as the principal focus of research, using deep learning (DL) designs for the analysis and measurement of these fluid regions (Yao et al., 2024). DL has significantly improved disease detection and diagnosis in medical image analysis (Choudhary et al., 2023). In 2016, the Google Brain project confirmed that its machine learning (ML) algorithms could effectively identify referable retinal pathologies, including DME and DR (Ong et al., 2021). The predominant method of DL for therapeutic image cataloging is supervised learning, which requires a large training data corpus of clearly labeled medical images. Generative adversarial networks (GANs) have been proposed as a new framework for unsupervised learning (Zheng et al., 2022). The transition from promising AI/ML models to their successful and practical clinical use fundamentally relies on efficient optimization. In addition to the fundamental training of neural networks, optimization methods are crucial for improving model performance, increasing interpretability, and ensuring deployment viability. The application across three principal dimensions—parameter optimization during model training to enhance predictive accuracy, feature selection to pinpoint the most significant biomarkers and augment model efficiency, and model optimization techniques designed to refine models for practical clinical integration and resource-limited settings—is the major area where optimization is used.

### 1.1 Research questions

1) What are the current trends and advancements?
2) What specific DL and ML models are employed most frequently?
3) How have optimization algorithms been integrated and used to enhance the performance of DL and ML models for DME-related tasks?
4) What problems, limitations, potential injustices, and practical obstacles have been observed in this regard?
5) What are the reported performance metrics and outcomes?
6) What are the existing research gaps and emerging trends or future directions?

## 2 Methods

This systematic review was conducted per the Joanna Briggs Institute methodology for systematic reviews of quantitative evidence. A review protocol was established to guide the review process and enhance transparency, outlining a comprehensive approach before the initiation of the assessment. This helps in evaluating the application of AI in ophthalmology, specifically in the diagnostic analysis of DME, and the types and purposes of optimization algorithms applied, which was the goal.

### 2.1 Search string

A comprehensive search method was devised to systematically identify pertinent literature on the application of computational intelligence in the detection and management of DME. The primary search term was formulated to identify research using AI methodologies for image-based analysis of DME, according to the search interface settings. The search criteria specifically integrated the primary topic “diabetic macular edema,” microaneurysms, with AI systems such as “deep learning” and “machine learning” in the abstract. To concentrate on image-based diagnoses, the phrases “Fundus Images” or “Retinal Images” were incorporated throughout all domains. Additionally, the engagement of optimization algorithms with the search keywords “optimization,” “optimizer,” and “soft computing” was delineated in the abstract, and the keyword “soft computing” was used in all fields. The phrases were deliberately combined using Boolean operators (AND, OR) to ensure significant relevance. Supplementary filters, such as “Full Text Online” and “Scholarly & Peer-Reviewed” publications, were used to enhance the search results for systematic reviews.

### 2.2 Search strategy

**Figure 1:**
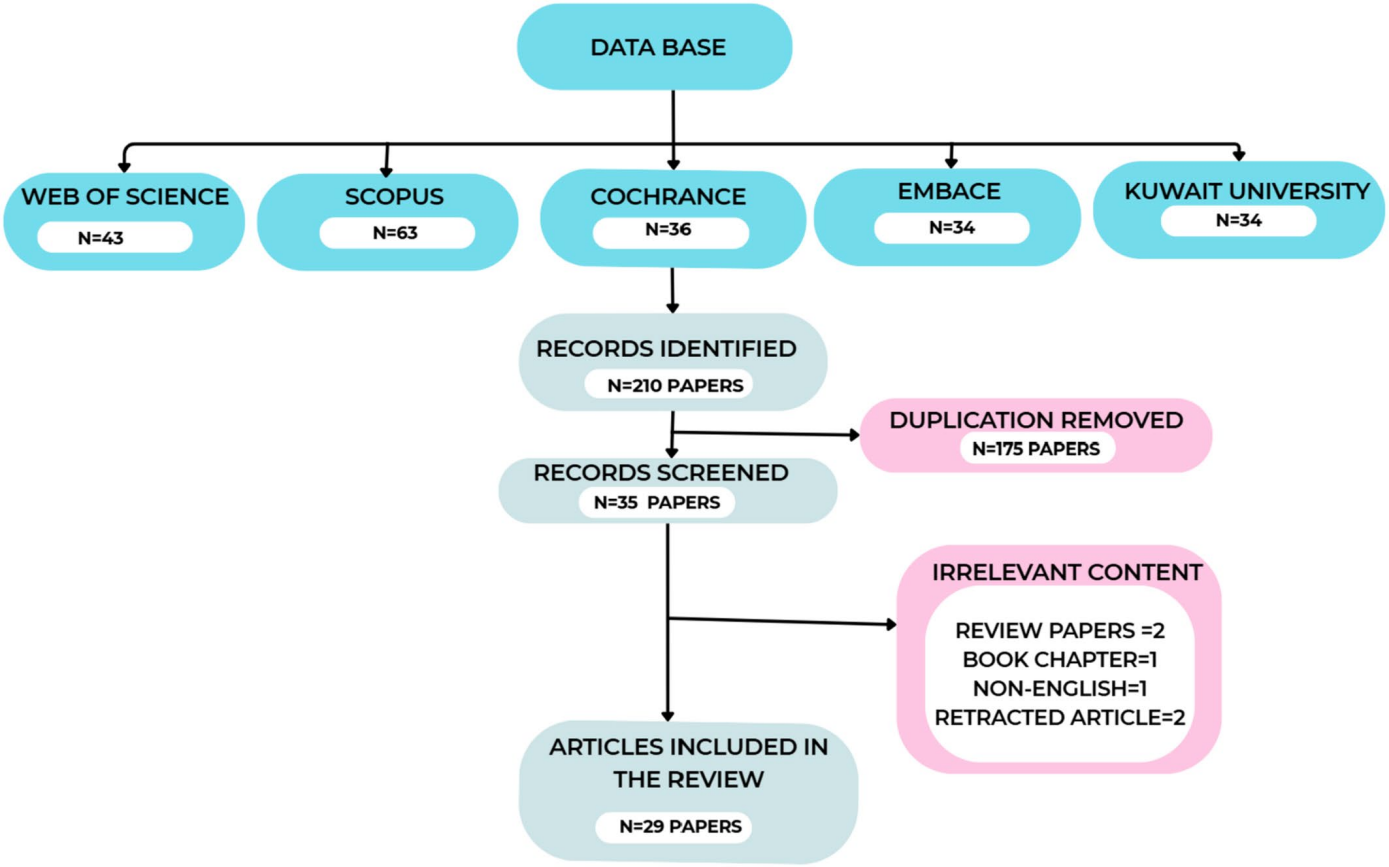
Flow chart of article assortment.

A comprehensive literature review was conducted over a decade, encompassing conference proceedings, journal articles, peer-reviewed sources, and catalogs such as Web of Science, Scopus, IEEE, Elsevier, and Science Direct journals. The Web of Science database provided 43 articles, the Scopus database 63 articles, the Coherence database 36 articles, Embase 34 results, and the Kuwait University library provided 34 articles; a total of 210 articles were identified with this search query using keywords across all areas, and we assembled a list of these articles. Upon refining our search string exclusively to abstracts and removing duplicates and survey articles, we acquired approximately 35 articles for our investigation. After removing duplicates and eliminating irrelevant content, a total of 32 collected articles remained. The withdrawn articles were excluded, and the study comprises 31 articles. We further excluded book chapters and were left with 29 articles for our review, encompassing publications concerning computer science and engineering. We focused on studies that address DME vis-à-vis DL and ML, adopting optimization techniques. Figure 1 indicates the process map of article assortment.

After a comprehensive database search, records were exported and deduplicated. A two-stage dual-independent screening process was employed to minimize bias. In the first, three reviewers, AM, KS, and BT, screened titles and abstracts against predefined inclusion and exclusion criteria, excluding clearly ineligible records and advancing potentially relevant ones. The second stage involved retrieving and assessing the full texts of advanced records. Discrepancies during the screening were categorized as “Include” or “Exclude.” Disagreements were discussed to reach a consensus; if unresolved, a fourth reviewer, SR, made the final decision, documented with reasoning. The entire process is illustrated in the flow diagram (Figure 1).

### 2.3 Selection and rejection principles

This systematic review focused on literature published from July 3, 2013, to July 3, 2025, to include the most recent studies. All detected records were exported to EndNote and subsequently deduplicated. The study selection process included a four-stage, independent screening method to reduce bias, per JBI and PRISMA recommendations. Four reviewers individually evaluated all titles and abstracts according to the established inclusion and exclusion criteria. Records that evidently failed to satisfy the qualifying requirements were excluded at this juncture. Exclusions were made for (1) non-English literature, (2) duplicate publications, (3) content irrelevant to ophthalmology or AI applications, (4) conference abstracts, (5) retracted articles, and (6) non-empirical works such as editorials, case reports, and commentaries. Table 1 presents the criteria for inclusion and exclusion.

**Table 1:** Selection and rejection principles.

Selection principles	Rejection principles
Diabetic macular edema	Non-English articles
OCT and fundus images	Duplicate publications
Articles with optimization application	Themes beyond the purview of ophthalmology or AI applications
Articles employing machine learning, deep learning, or hybrid methodologies in the sorting or estimation stages	Conference abstracts
Articles encompassing measurements of accuracy, sensitivity, recall, specificity, AUC, and *F*_1_-score	Retracted articles

### 2.4 PICO framework

**Figure 2:**
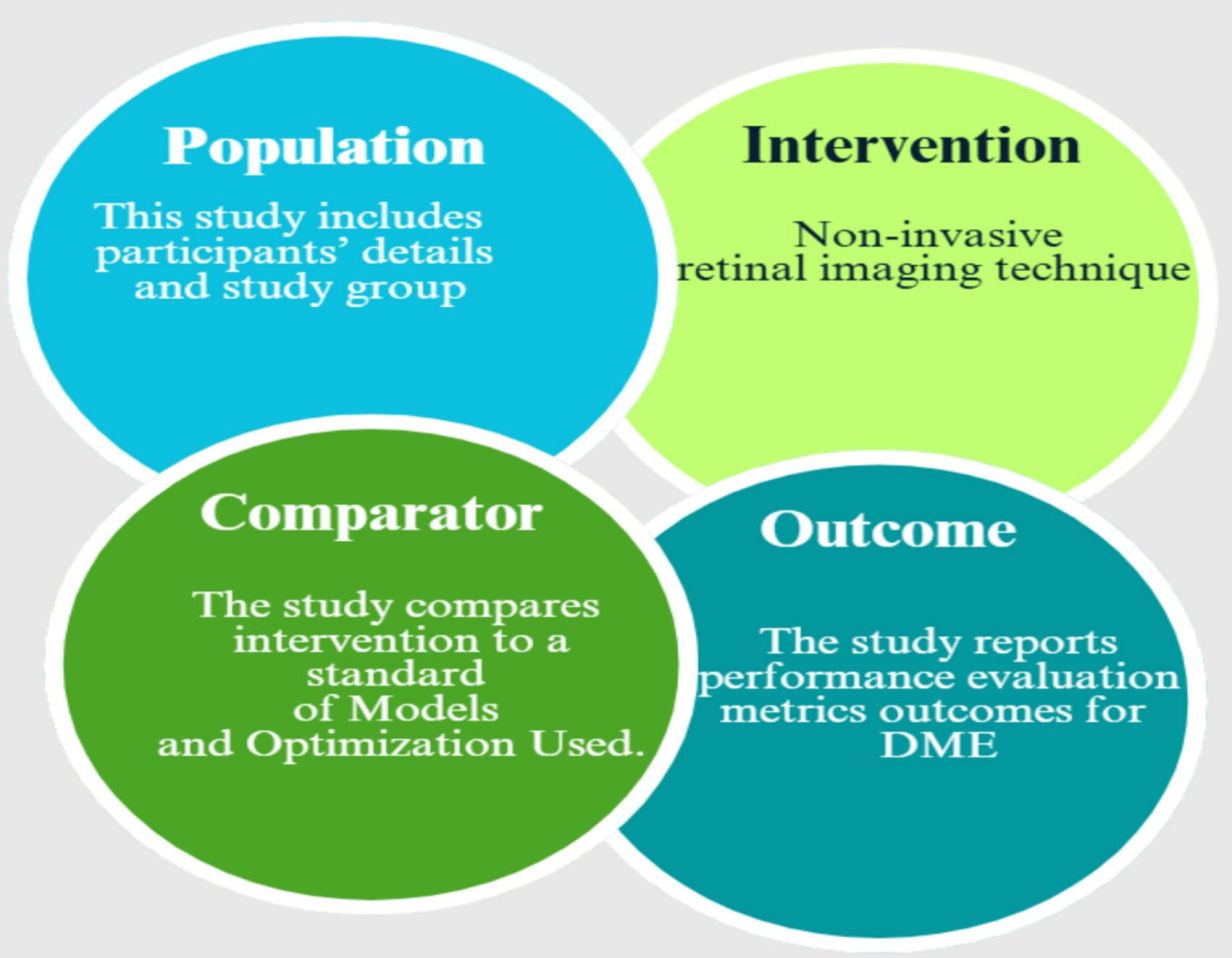
PICO framework.

This study concentrated on DME, microaneurysms, DL, ML, optimization, analyzing models, and soft computing as the principal technologies under consideration. Performance metrics, including accuracy, precision, recall, *F*_1_-score, and AUC score, were recorded. The specific domains of consideration are computer science and engineering. The PICO framework thoroughly assesses each work, as shown in Figure 2.

### 2.5 Research protocol and registration

The protocol for this systematic literature review was created *a priori* and documented in compliance with the PRISMA criteria. Although protocol registration is not a conventional prerequisite for reviews in the engineering field, we documented the entire technique to ensure transparency and rigor. The conclusive protocol is retrospectively registered with the Open Science Framework (OSF) Registries and is accessible via the unique identifier osf.io/qh4r3.

## 3 Results

**Figure 3:**
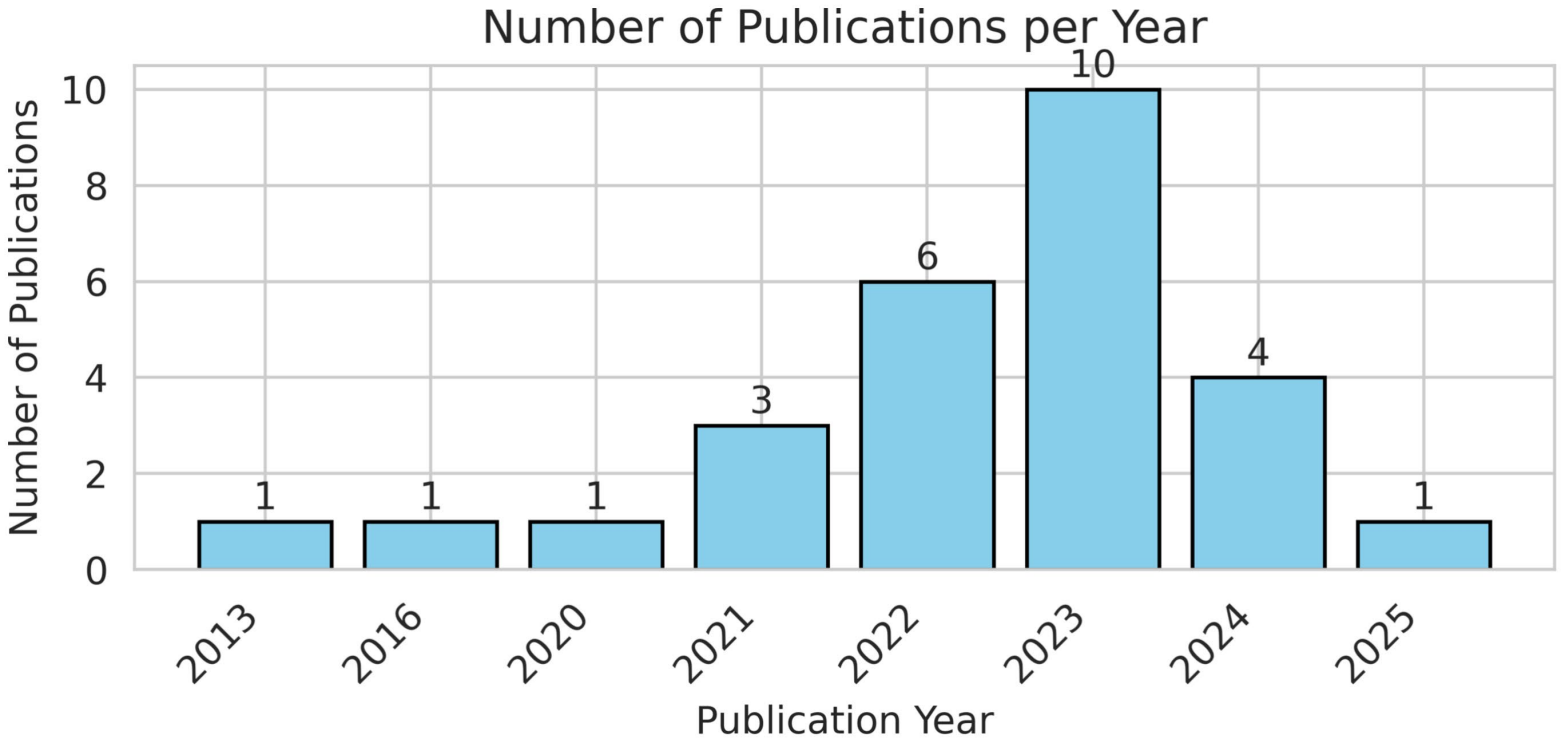
Publications per year.

**Figure 4:**
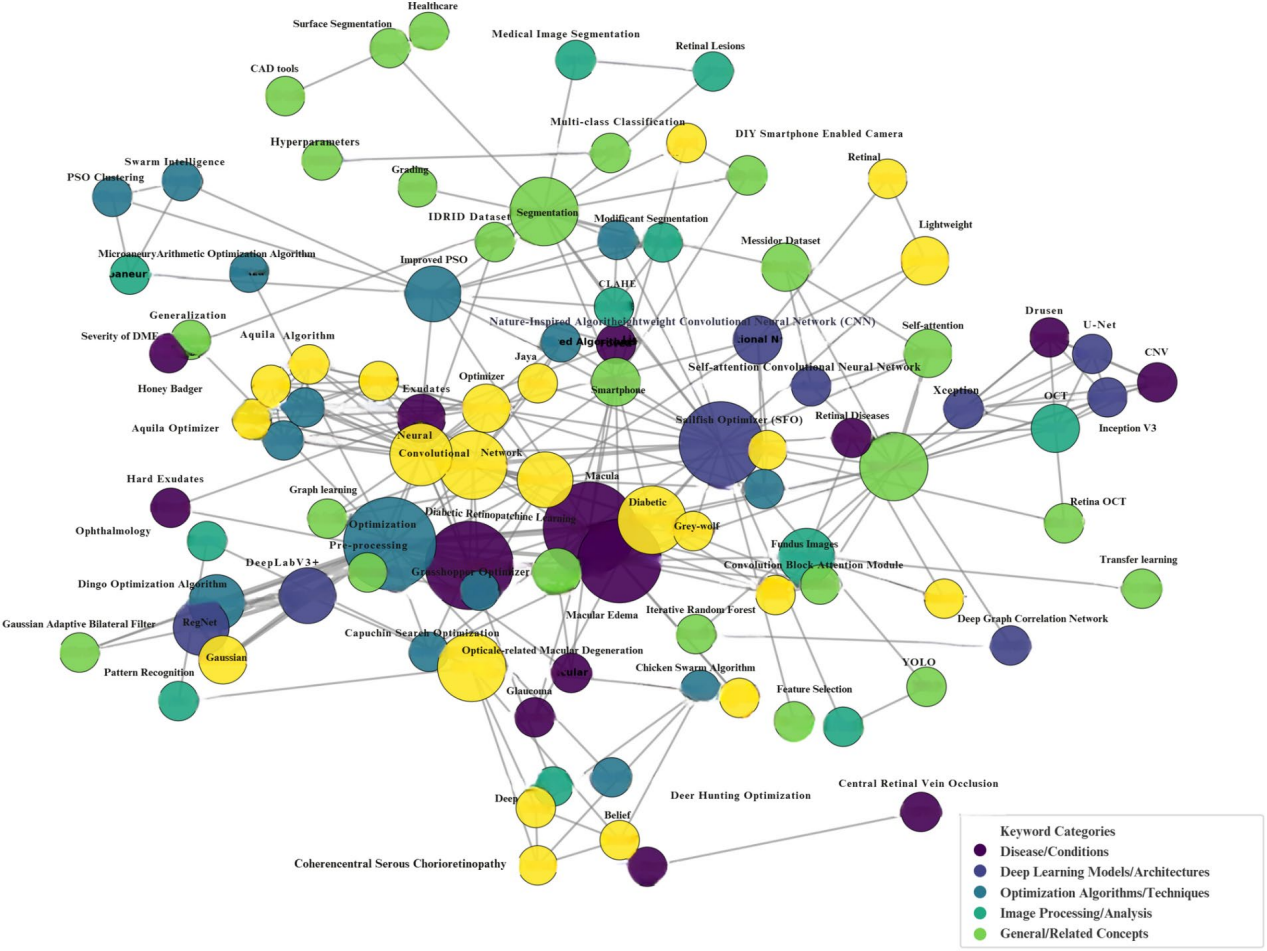
Coherence diagram of keywords.

The bar chart, entitled “Number of Publications per Year,” illustrates the annual distribution of research output from 2013 to 2025. Initially, the research domain demonstrated limited engagement, with a solitary publication noted in the years 2013, 2016, and 2020. A perceptible increase commenced in 2021, culminating in three publications, which then surged in the following years. The apex of research productivity was reached in 2023, with 10 articles published. Nevertheless, this increase was succeeded by a decrease, with four publications in 2024 and just one publication projected for 2025. As of July 2025, the 2025 figure may not represent the total publication volume for the year. The graphic delineates a clear lifespan of research interest: a subdued beginning, a phase of accelerated growth and peak engagement, succeeded by a possible decline or, for the latest year, an incomplete tally. Figure 3 illustrates the number of publications per year. Figure 4 presents the coherence diagram for all keywords used by different authors in the literature. It consists of diseases with the specific keywords of Diabetic Retinopathy, Hard Exudates, Retinal Diseases, Exudates, Fovea, Macula, Severity of DME, Glaucoma, Macular Edema, CNV, Drusen, Age-related Macular Degeneration, Branch Retinal Vein Occlusion, Central Retinal Vein Occlusion, and Central Serous Chorioretinopathy. DL Architectures, such as CNN, Lightweight CNN, Self-attention Convolutional Neural Network, Auto-Metric Graph Neural Network, Deep Belief Network, DeepLabV3+, RegNet, U-Net, InceptionV3, Xception, Deep Graph Correlation Network, Optimization Algorithms/Techniques, Image Processing, such as Image Retrieval, Fundus Images, Lesions Detection, Fuzzy Image Processing, Microaneurysms, Contrast Enhancement, CLAHE, Semantic Segmentation, Image Processing, Image Analysis, Medical Image Segmentation, Retinal Features, Retinal Lesions, Ophthalmology, OCT, Pattern Recognition, Image Segmentation Techniques, and General/Related Concepts such as YOLO and Messidor Dataset are included. Table 2 presents a summary of the reviewed articles.

**Table 2:** Summary of articles.

Author (year)	Aim of study	Dataset	Imaging	Study group	Model used	Outcomes	Limitations
Santos et al. (2022)	Detect fundus lesions using YOLO	OIA-DDR	Fundus images	Not specified	YOLO	Accuracy: High, Sensitivity: Good, Specificity: Good *F*_1_-score: 0.2521 mAP = 0.1540	Data imbalance, lack of dataset diversity
He et al. (2021)	To develop an integrated context for designing coating surface segmentation in OCT images	Public datasets (HC and DME)	Optical coherence tomography	35 subjects (14 HC, 21 PwMS)	Fully convolutional network (FCN)	MAD: 6.70, RMSE: 4.51	Limited to 2D B-scans, single dataset reliance
Zhang et al. (2022)	To predict photographic acuity after anti-VEGF treatment in DME patients using machine learning	Department of Ophthalmology, Qilu Hospital 281 eyes	Optical coherence tomography (OCT)	281 eyes, mean	Ensemble model (LR + RF)	MAE: 0.137 logMAR, MSE: 0.033 logMAR	Small sample size, single dataset, manual data extraction
Gupta et al. (2022)	To develop an enhanced fusion ML method for smartphone-built DR recognition	APTOS-2019, EyePacs	DIY smartphone camera	35,126 retina images (both eyes)	NN-DCNN-SSD	Accuracy: 98.9% (EyePacs), 99% (APTOS-2019); Sensitivity: 97.4, 97.5%; Specificity: 99.3%	Single dataset reliance
Khan et al. (2023)	To develop a cross-deep learning technique for OCT image cataloging	OCT images from Soonchunhyang University Bucheon Hospital	Optical coherence tomography	2,998 images	DenseNet-201, InceptionV3, ResNet-50	Accuracy: 99.1% (with ACO), 97.4% (without ACO)	Single dataset reliance, potential labeling bias
Khan et al. (2024)	To develop a lightweight model for retinal image segmentation	DRIVE, STARE, IDRiD	Fundus imaging	40 (DRIVE), 20 (STARE), 81 (IDRiD)	LMBF-Net	*n*: 83.48%, Sp: 98.77%, Acc: 96.97%, *F*_1_: 98.46%, AUC: 84.22%	Limited data, single dataset
Purna Chandra Reddy and Gurrala (2022b)	To enhance the classification accuracy of DME and DR using OHGCNet	IDRiD	Fundus images	Not specified	OHGCNet	DME: 99.03%, DR: 98.31%, joint DR-DME: 98.67%	Single dataset, computational complexity
Wang et al. (2016)	To develop a CAD model for AMD and DME detection	SD-OCT dataset	OCT	45 participants (15 AMD, 15 DME, 15 Normal)	SMO, SVM	Accuracy: 99.3%, Sensitivity: 99.3% (AMD), Specificity: 99.6% (Normal), AUC: 0.996	Single dataset limits generalizability
Purna Chandra Reddy and Gurrala (2022a)	Combined arrangement of DR and DME	IDRiD	Fundus pictures	516 images	ResNet50, MGWO	Accuracy: DR 96.0%, DME: 93.2%, Joint: 92.23%	Single dataset, potential bias
Purna Chandra Reddy and Gurrala (2023)	To classify joint DR-DME using deep learning	IDRiD	Color fundus images	413 training images, 103 testing images	JDD-Net	DR: 99.53%, DME: 99.1%, Joint: 99.01%	Single dataset, class imbalance
Reddy and Soma (2025)	Early detection and classification of DME	OCTIRD	OCT	500 + samples	SIH + HBAO-based deep CNN	Accuracy: 91.2%, Sensitivity: 91.7%, Specificity: 91.8%	Limited generalizability
Naik et al. (2023)	Develop an ensemble deep learning model with attention for OCT-based eye disease prediction	Kermany et al. (2018) (207,103 images, 4 classes)	OCT	500 images/class for training, 1,000 test images (250/class)	U-Net (segmentation), InceptionV3, Xception (ensemble with self-attention)	U-Net: Acc 94.69%, IOU 84.08%; Ensemble: Acc 96.69%, Precision 96.71%, Recall 96.69%, *F*_1_ 96.69%	Small annotated set, single dataset, no external validation, hardware limits
Karthik and Mahadevappa (2024)	Optimize deep learning training for OCT imaging	Public dataset (Mendeley Data)	Optical coherence tomography	84,495 images (4 classes)	AlexNet, VGG, ResNet, RetiNet, AOCT-NET, DeepOCT, Octnet	Accuracy improvement: 0.28 to 12.6%, Training epochs reduced: 4.35 to 58.27%	Limited generalizability due to a single dataset
Mugada and Lakshmi (2025)	To design an efficient DME grading system	IDRiD, Kaggle	Fundus Images	516 images, various ages	ATL-MobileNet, Dil-M TransUnet++	Accuracy: 92.38%, Sensitivity: 85.89%, Specificity: 96.00%	Limited generalizability, reliance on a single dataset
Navaneethan and Devarajan (2024a)	Enhance diabetic retinopathy detection	Fundus image dataset	Fundus imaging	420 images	CNN, GAN	Accuracy: 98.8%, Precision: 98.7%, Recall: 96.5%, Specificity: 97.1%, *F*_1_-measure: 98.2%, Kappa: 95%	Limited generalizability due to a single dataset
Ahmed et al. (2025)	Early detection of macular edema	Duke OCT dataset	OCT	45,000 images	DIO-RegNet	Accuracy: 99.44%, Specificity:95.27% Precision: 93.77%, Recall: 97.31% *F*_1_-score: 96.47%	Limited dataset diversity
Rajagopalan et al. (2021)	To classify retinal disorders using CNN from OCT images	Mendeley dataset	OCT	12,000 images (3,000 per class)	CNN	Accuracy: 97.01%, Sensitivity: 93.43%, Specificity: 98.07%	Single dataset, potential bias
Krishna and Rao (2023)	To develop a DL model for multi-class classification of DED	DRISHTI-GS, Messidor-2, etc.	Fundus Imaging	1,748 images	DeepID3 net	Accuracy: 99.23%, Sensitivity: 98%, Precision: 98.13%, Recall: 98%, *F*_1_-score: 98.3%, Specificity: 98.28%	Limited dataset diversity
Reddy et al. (2023)	Develop a robust DME classification method using DBN + MCSA	OCT images (source not specified)	OCT	Not specified	DBN (optimized by MCSA + Firefly)	Accuracy: 97.15%, Sensitivity: 97.8%, Specificity: 97.8%	Dataset source/split unclear, no external validation, limited demographic info
Aurangzeb et al. (2021)	Enhance the contrast of fundus images to improve deep learning model performance	DRIVE, STARE	Fundus imaging	Not specified	Deep learning models (e.g., CNNs)	Sensitivity: 0.8315 (DRIVE), 0.8433 (STARE); Specificity: 0.9750 (DRIVE), 0.9760 (STARE); Accuracy: 0.9620 (DRIVE), 0.9645 (STARE)	Limited dataset diversity, need for external validation
Jasper Gnana Chandran et al. (2023)	To propose an AGNN enhanced with CSOA aimed at grading DR and DME	ISBI 2018 IDRiD, Messidor	Fundus imaging	516 images (IDRiD), 1,200 images (Messidor)	AGNN-CSO-DR-DME	Accuracy: 99.57, 97.28, 96.34% (IDRiD); Higher accuracy on Messidor; *F*-measure values significantly higher than existing methods; Lower execution time compared to existing methods	Limited to specific datasets; Potential injustices not addressed
Sreejini and Govindan (2013)	Automatic unsupervised grading of DME severity in color fundus images	MESSIDOR (100 images)	Color fundus photography	100 images	PSO-based multilevel thresholding, mathematical morphology	Sensitivity: 82.5%, Specificity: 100%, Accuracy: 93%	Optic disc vs. exudates confusion, missed faint/small exudates, no external validation
Elwin et al. (2022)	To develop an Ar-HGSO-based deep learning model for DR detection and severity classification	IDRID, DDR	Color fundus images	Not specified	DCNN, ShCNN	Testing Accuracy: 0.9142, Sensitivity: 0.9254, Specificity: 0.9142 Testing Accuracy: 0.9142, Sensitivity: 0.9254, Specificity: 0.9142	Limited dataset, potential overfitting, lack of external validation
Lu et al. (2023)	To develop a lightweight CNN for joint classification of DR and DME	Messidor dataset (3,028 images)	Fundus photography	3,028 images, age not specified	ShuffleNet V2	Accuracy: 96.66%, Precision: 97.01%, Recall: 96.86%, Specificity: 99.33%, *F*_1_-score: 96.63%	Class imbalance, limited dataset, image-level supervision
Devi and Karthikeyan (2024)	To propose a unified grading solution for DR and DME using SACNN optimized with AOA	Messidor, ISBI 2018 IDRiD	Fundus Imaging	1,488 images (50% training, 50% test)	SACNN-AOA-DR-DMEG	Accuracy: 98.95%, Precision: 98.87%, Recall: 98.97%, *F*_1_-measure: 98.97%, Computation time: 80 s	Limited external validation, reliance on a single dataset
Zeng et al. (2021)	To develop a multi-scale self-attention system for DR image reclamation	Kaggle DR dataset	Fundus imaging	35,088 images (28,086 training, 7,022 testing)	Multi-scale self-attention network with ResNet-50	mHR (*k* = 5): 0.6971, mAP (*k* = 5): 0.7259	Limited external validation, reliance on a single dataset
Rawat and Kumar (2024)	To recommend a hybrid DL style for improved generalizability in diabetic retinopathy detection	DRIVE dataset	Fundus imaging	40 RGB retinal images (70% training, 20% validation, 10% testing)	CNN with SAJOA	Accuracy: 96.51%, *F*_1_-score: 87.72%, Sensitivity: 98.91%, Specificity: 97.91%, Precision: 85.89%	Limited external validation, reliance on a single dataset
Chincholi and Koestler (2023)	To assess the routine of deep learning models for detecting DR and DME	DIARETDB1	Color fundus images	89 images (84 NPDR, 5 normal)	Faster R-CNN, Mask R-CNN	Accuracy: 99.34% (Mask R-CNN), 99.22% (Faster R-CNN); Sensitivity: 97.5% (Mask R-CNN), 97.37% (Faster R-CNN); Specificity: 96.6% (Mask R-CNN), 96.49% (Faster R-CNN)	Limited dataset, lack of detailed annotations
Bhimavarapu and Battineni (2022)	To detect microaneurysms for the early diagnosis of DR	DIARETDB0	Digital fundus images	130 images (various severity levels)	Improved PSO, Fuzzy Logic	Accuracy: 99.9%; Sensitivity: 99.8%; Specificity: 99.1%	Limited dataset, lack of detailed annotations

**Figure 5:**
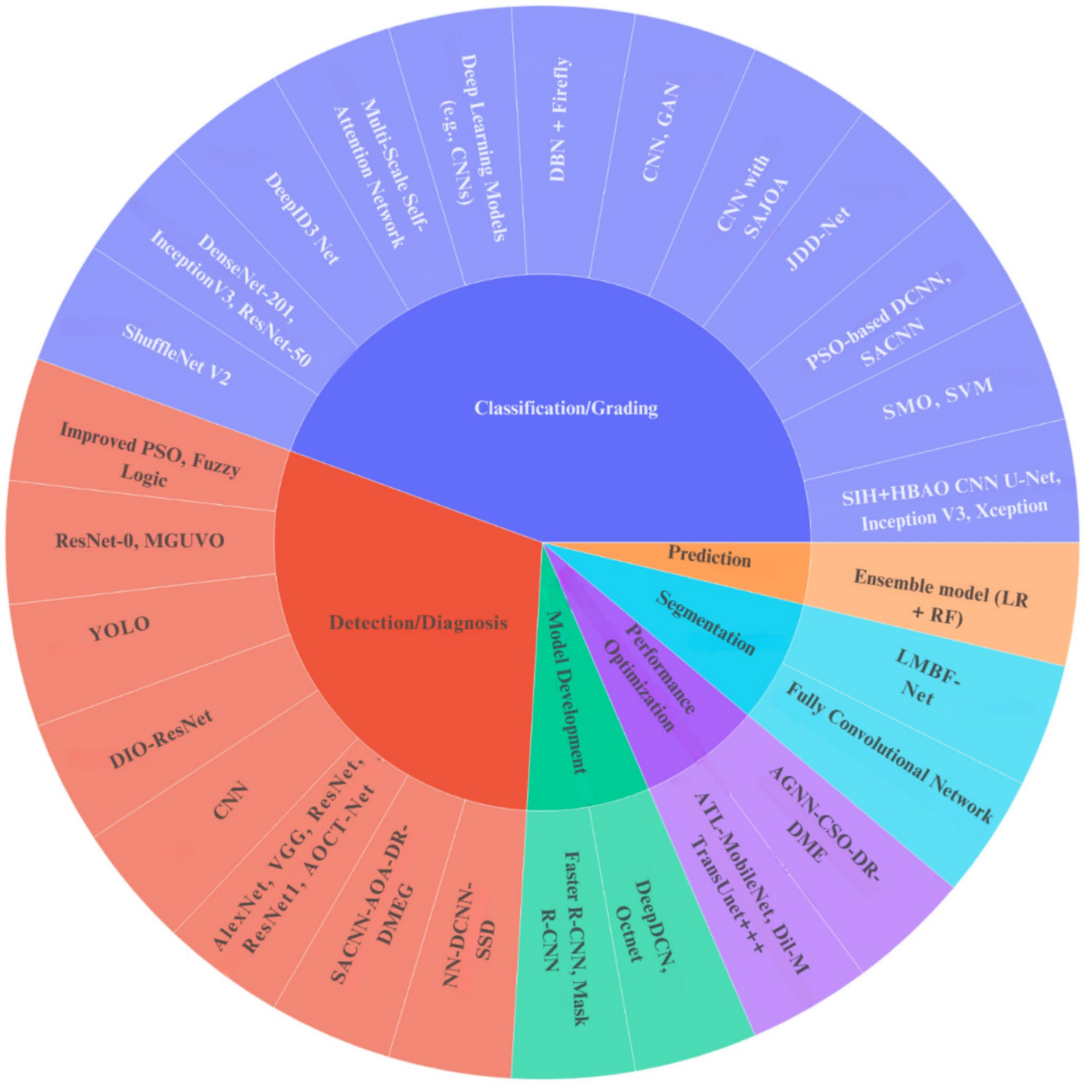
Sunburst diagram for the model used and purpose.

The sunburst diagram in Figure 5 illustrates the correlation between the ML models employed and the principal objectives of the investigations. The outer circle of the diagram illustrates several “Models Used,” including YOLO, FCN, DenseNet-201, and enhanced PSO. Each model then extends to an inner ring that delineates the “Categorized Aim” of the study, such as “Detection/Diagnosis,” “Classification/Grading,” “Segmentation,” “Prediction,” “Performance Improvement/Optimization,” or “Model Development/Framework.” The dimensions of each segment in the diagram reflect the frequency with which a specific model was employed for a particular research objective, providing a succinct overview of prevalent applications for various models.

### 3.1 Comparative analysis

The comparative examination of performance measures indicates significant efficacy among specialized models while also exposing methodological flaws and ongoing issues in the field. The Lightweight Multipath Bidirectional Focal Attention Network-Net, achieving a peak accuracy of 99.63% and an *F*_1_-score of 95.00, exemplifies the forefront of robust image segmentation, indicating that highly specialized architectures tuned for feature localization produce nearly flawless outcomes. The high classification accuracy of 98.91% attained by Dai et al. (2024) corroborates that ML and DL models, especially those using specialized Honey Badger Aquila Optimization, can accomplish precise classification of DR stages. A substantial disparity is observed in the shift to intricate detection tasks, as demonstrated by the YOLO-based method, which reports a mean average precision (mAP) of merely 15.4% (Toto et al., 2024). The pronounced disparity (99.63% accuracy vs. 15.4% mAP) does not indicate a deficiency in the model type; instead, it underscores the intrinsic challenges of real-time, multi-object detection, which necessitates accurate bounding box localization in conjunction with classification, especially for small or subtle lesions across diverse fundus images. The distinction highlights a significant methodological discovery: algorithms designed for the classification and segmentation of predefined regions, where metrics, such as a high AUC of 1.0, are attainable, encounter difficulties when addressing the more complex, broader issue of raw lesion detection. Future engineering research must focus on formulating robust optimization strategies to enhance mAP performance in detection models, thereby reconciling the disparity between highly accurate specialist systems and practical, real-time diagnostic tools for joint DR-DME classification. The Optimized Hybrid Machine Learning Method attained 99% accuracy in DR detection, demonstrating significant efficacy in smartphone applications.

### 3.2 Optimization techniques

**Figure 6:**
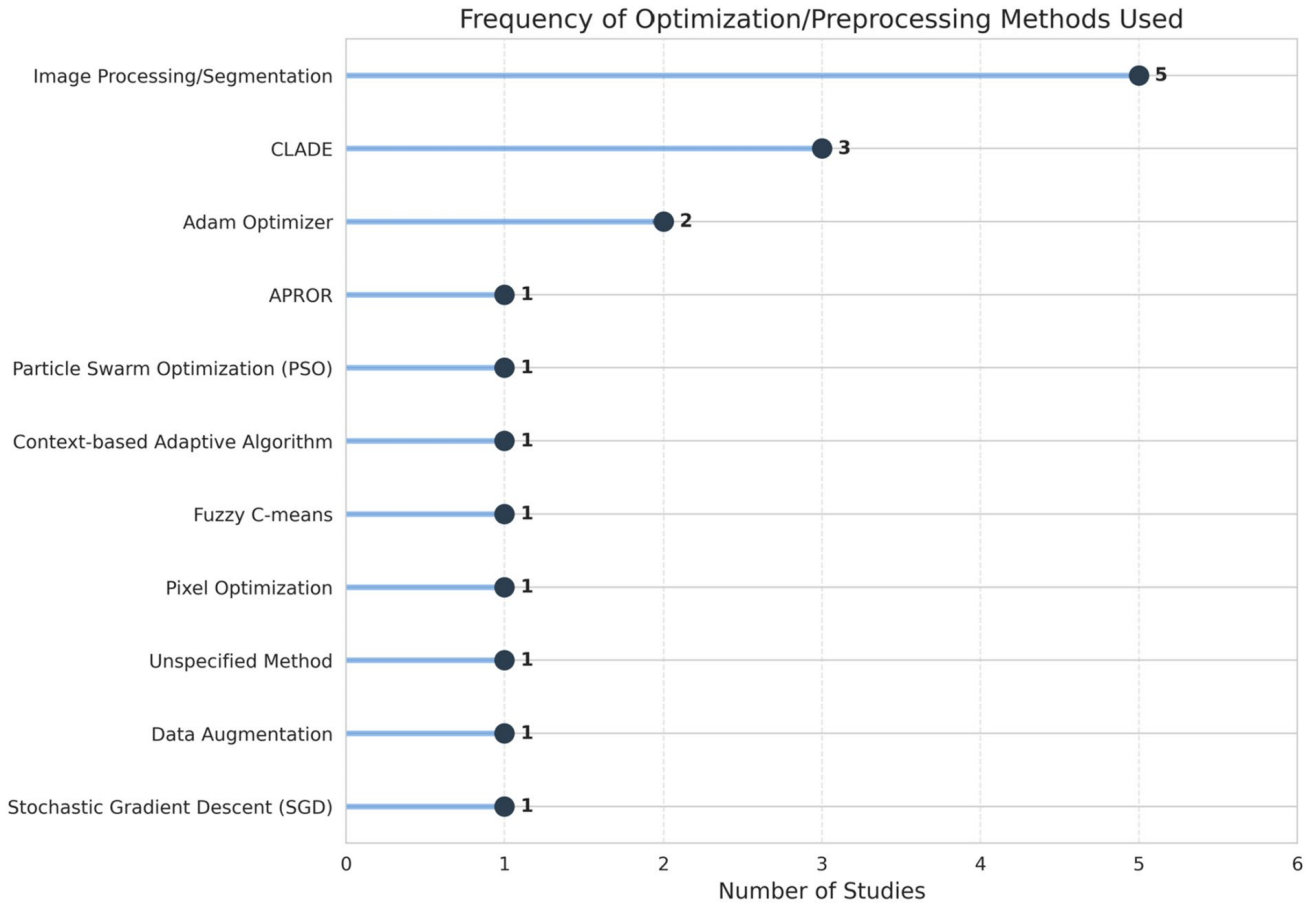
Frequency diagram of method used.

**Figure 7:**
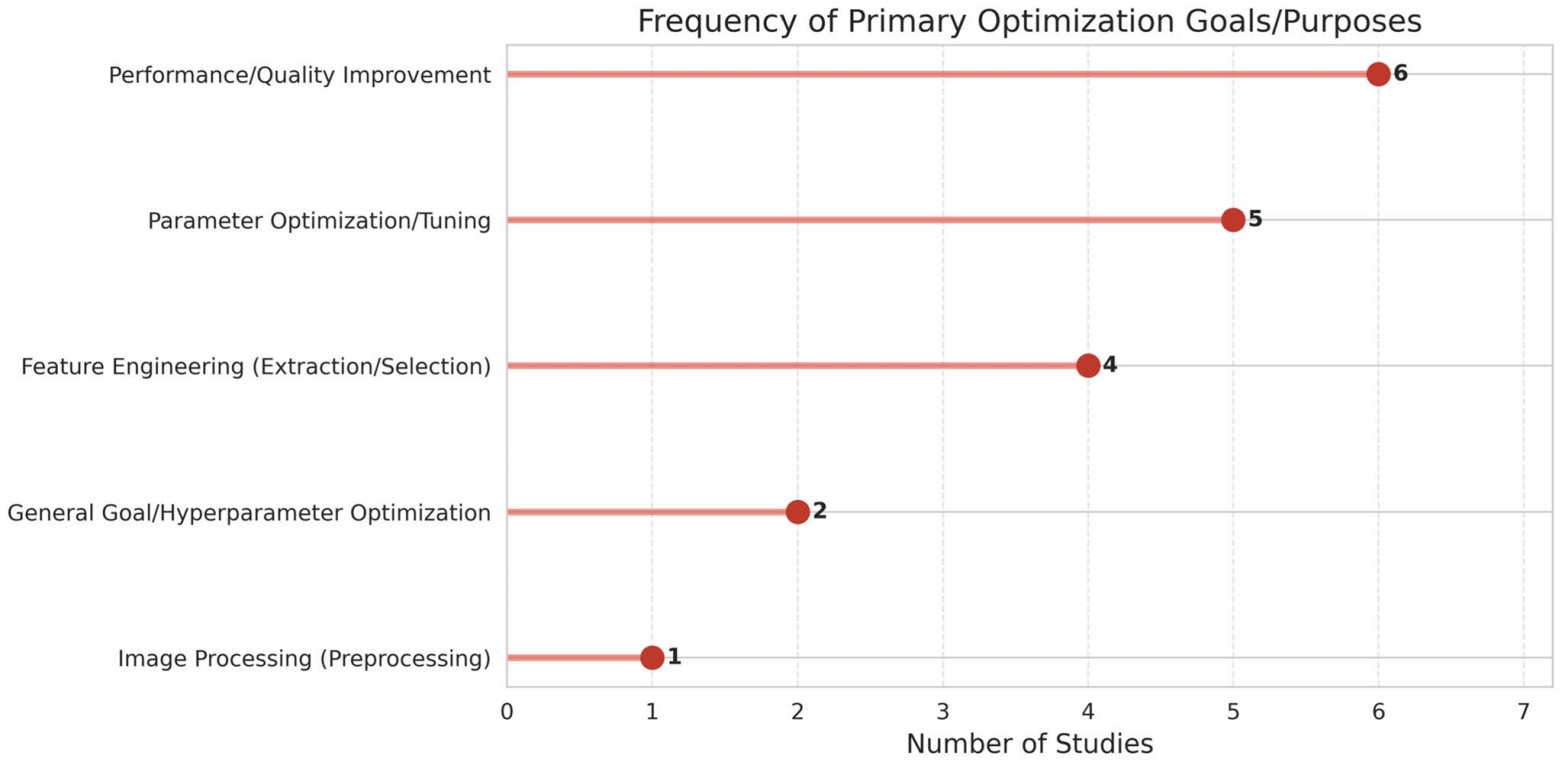
Frequency diagram of primary optimization.

Table 3 illustrates the diverse types of optimization algorithms used in survey learning. The usage of preprocessing and optimization algorithms is shown in Figures 6, 7.

**Table 3:** Optimization used.

Author (year)	Preprocessing	Optimization used	Purpose
Santos et al. (2022)	—	Stochastic gradient descent (SGD)	Optimizing batch size, epochs, and erudition rate
He et al. (2021)	Contrast Enhanced	Adam optimizer	Optimizing hyperparameters such as an initial learning rate of 10^–4^ and weight decay of 10^–4^
Zhang et al. (2022)	—	Grid search	Optimizing model parameters
Gupta et al. (2022)	CLAHE (contrast limited adaptive histogram equalization)	Life choice-based optimizer (LCBO) and Social ski-driver (SSD)	Enhance feature selection, weight optimization
Khan et al. (2023)	Pixel variations	Ant colony optimization (ACO)	Feature selection algorithm
Khan et al. (2024)	Patch-based implementation	Adam optimizer, filter optimization	Training and optimizing the number of filters
Purna Chandra Reddy and Gurrala (2022b)	Not specified	Modified deer hunting optimization algorithm (MDHOA)	For the finest feature
Wang et al. (2016)	Image preprocessing to handle quality issues	Correlation-based feature subset	Feature optimization and avoiding overfitting
Purna Chandra Reddy and Gurrala (2022a)	Data augmentation (rotation, scaling, cropping)	Modified Grey-Wolf Optimizer (MGWO)	Feature selection and enhancing classification performance
Purna Chandra Reddy and Gurrala (2023)	Image augmentation techniques	IRF-Net	Optimal feature selection
Reddy and Soma (2025)	Gaussian filtering	HBAO algorithm	Improve convergence speed
Naik et al. (2023)	Normalization, resizing (496 × 496 for U-Net, 299 × 299 for classifiers)	Self-attention integration	Weight adjustment
Karthik and Mahadevappa (2024)	No preprocessing to maintain uniformity	Entropy-based	Early stopping
Mugada and Lakshmi (2025)	Median filtering, DWT	RVE-HSOA algorithm	Weights in the MobileNet
Navaneethan and Devarajan (2024a)	Attenuation of image difference, intensity conversion, denoising, disparity enhancement	Crossover Grasshopper optimizer algorithm (GOA) and Salp swarm algorithm (SSA)	Improve the optimization process of the model, regulate the search domain, and avert convergence to local optimal solutions
Ahmed et al. (2025)	Gaussian adaptive bilateral filter	Dingo optimization algorithm	Feature selection and classification
Rajagopalan et al. (2021)	Speckle noise reduction, resizing	Random search Stochastic gradient descent (SGD)	Batch size: Optimized to 100 Epochs: Set to 50 Dropout rate: Adjusted to 0.2 Minimizing the loss function
Krishna and Rao (2023)	Mathematical morphology for contrast enhancement	Flower pollination optimization algorithm	Network optimization
Reddy et al. (2023)	Active contour segmentation, FFT, LGP, layer-specific features	Hybrid chicken swarm and firefly algorithm	Model optimization
Aurangzeb et al. (2021)	CLAHE optimized by MPSO	Modified particle swarm optimization (MPSO)	Tuning the parameters of CLAHE
Jasper Gnana Chandran et al. (2023)	APPDRC filtering method for noise removal and feature extraction	Capuchin search optimization algorithm (CSOA)	To optimize the AGNN parameters
Sreejini and Govindan (2013)	Median filtering, color normalization (histogram specification), resizing, green channel extraction	Particle swarm optimization (PSO)	For effective segmentation
Elwin et al. (2022)	Median filtering, ROI extraction, EWKPC for lesion segmentation	Autoregressive-Henry gas sailfish optimization (Ar-HGSO)	To enhance prototypical performance
Lu et al. (2023)	Contrast-limited adaptive histogram equalization, median filtering, image resizing	Stochastic gradient descent (SGD)	Optimizing learning rates
Devi and Karthikeyan (2024)	APPDRC for noise reduction	Arithmetic optimization algorithm	Improve its performance metrics
Zeng et al. (2021)	Images resized to 224 × 224 × 3 Content-based image retrieval (CBIR)	Triplet loss	This method reduces the proximity of like images while increasing the separation from disparate ones
Rawat and Kumar (2024)	CLAHE, data augmentation (optical distortion, flips, rotations)	Self-adaptive Jaya optimization algorithm (SAJOA)	Optimize the hyperparameters of the CNN
Chincholi and Koestler (2023)	Labelme for annotations, COCO format conversion	Stochastic gradient descent (SGD)	Model training
Bhimavarapu and Battineni (2022)	Fuzzy image enhancement, PBPSO for segmentation	Discrete particle swarm optimization (PSO) Probability-based PSO (PBPSO)	Clustering and segmentation Optimizing the clustering process

### 3.3 Hyperparameter optimization

Hyperparameter optimization (HO) entails modifying the external, predetermined variables of the training process in optimizing the learning rate and number of layers, with algorithms such as Jaya or Ar-HGSO to enhance the model’s prediction performance. Conversely, model optimization emphasizes inherent architectural or data-processing improvements; creating a lightweight CNN to reduce computational complexity or using a Modified PSO for Contrast Enhancement to enhance the quality of input features supplied to the model, thus increasing the convergence rate and deployment viability are examples. This implies that meta-heuristic techniques are not only “enhancing performance” in a general sense but are explicitly used for either maximizing metrics (HO) or improving efficiency and feature quality (model optimization). The analysis highlights the crucial importance of optimization in enhancing model efficiency and convergence speed, a fundamental engineering consideration for clinical applications. The LMBF-Net demonstrated higher training efficiency, achieving convergence 2.7 times more rapidly than prior models. This significant acceleration is attributed to both the architecture and the integrated multipath bidirectional focal attention mechanism, which effectively optimizes the feature space and loss landscape, thus transforming structural innovation into accelerated learning and improved accuracy. The successful application of Honey Badger Aquila Optimization and similar bio-inspired techniques in research like joint DR-DME classification demonstrates a significant trend: the shift toward customized, meta-heuristic optimization strategies to rectify the limitations of traditional gradient descent methods. These nature-inspired algorithms are designed to circumvent local minima and traverse complex hyperparameter spaces more effectively, leading to improved overall model performance and a more efficient path to the optimal solution.

**Figure 8:**
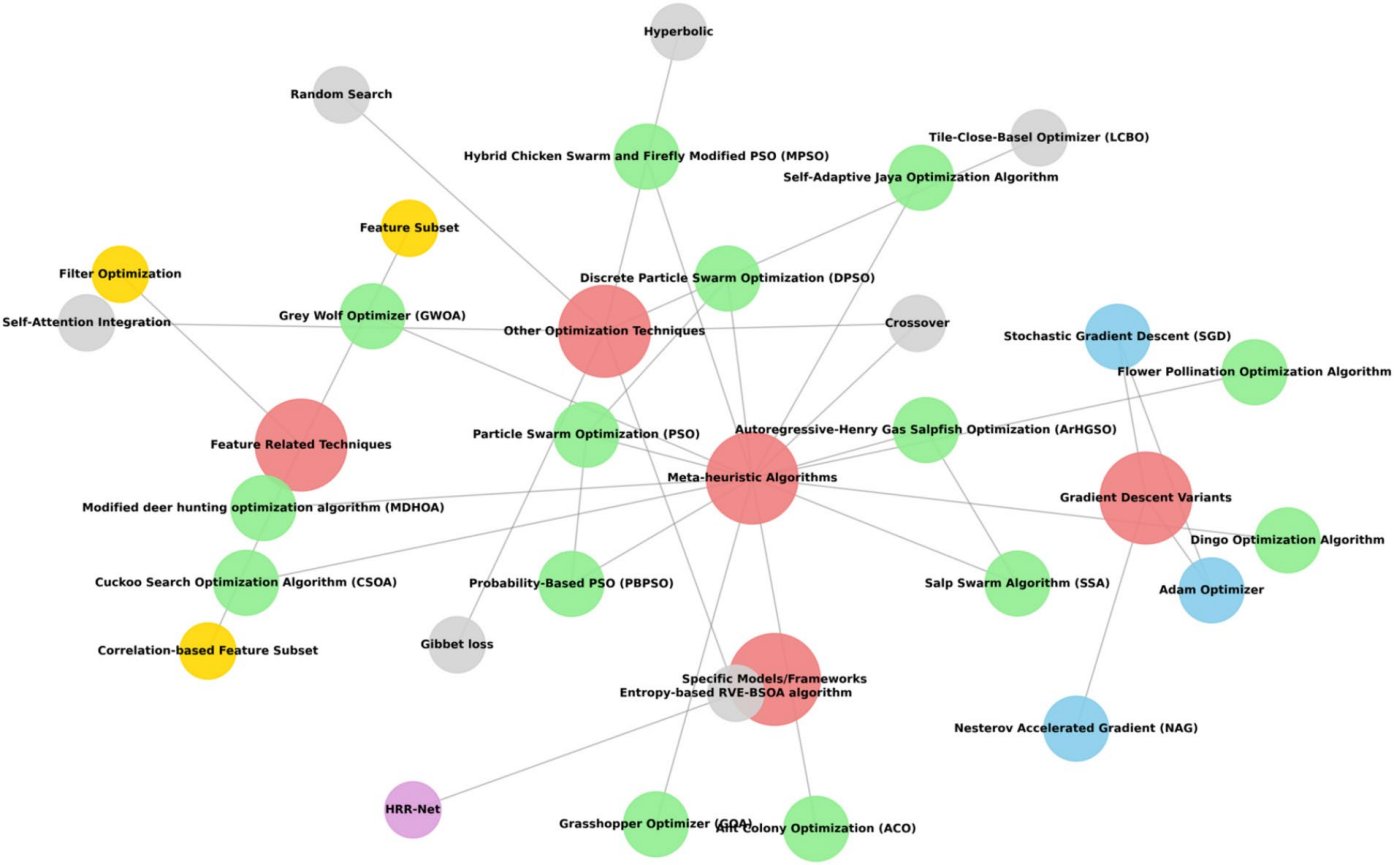
Network diagram for optimization algorithm.

Figure 8 indicates the network diagram of the different optimization algorithms used. The network diagram consists of meta-heuristic algorithms such as, Dingo Optimization Algorithm, Flower Pollination Optimization Algorithm, Hybrid Chicken Swarm and Firefly Modified PSO (MPSO), Cuckoo Search Optimization Algorithm (CSOA), Particle Swarm Optimization (PSO), Discrete Particle Swarm Optimization (DPSO), Probability-Based PSO (PBPSO), Ant Colony Optimization (ACO), Modified Deer Hunting Optimization Algorithm (MDHOA), Grey Wolf Optimizer (GWOA), Grasshopper Optimizer (GOA), Slap Swarm Algorithm (SSA), Autoregressive-Henry Gas Salpfish Optimization (ArHGSO), and Self-Adaptive Jaya Optimization Algorithm. Other optimization techniques include Random Search, Tile-Close-Basel Optimizer (LCBO), Self-Attention Integration, Entropy-based RVE-BSOA algorithm, Crossover, Hyperbolic, Gibbet loss, Feature Related Techniques such as Filter Optimization, Correlation-based Feature Subset, and Specific Models such as HRR-Net.

## 4 Modeling analysis

The publication year, author, model used, optimization algorithm, dataset, performance metrics, limitations, and findings related to optimization are the specific data points to be extracted from each paper. We used a data extraction form to systematically extract all relevant information from each article. Criteria for experimental design, validation, dataset size, and reproducibility assess the methodological quality and risk of bias (RoB) of individual studies. Descriptive statistics are conducted in our studies to analyze and combine to answer the research questions. The results will be presented in both table and graph formats. All conducted searches were independently peer-reviewed by a second analyst, and any differences were resolved through discussion or the involvement of a fourth author. The peer evaluation of the Electronic Search Strategies checklist elements guided the search strategy peer evaluation. The RoB assessment was assessed to ensure that the authors’ conclusions and findings were grounded in the most reliable evidence and that any potential sources of bias in the data were recognized. The quality of the studies was assessed by three writers (AM, KS, and BT). Disputes were settled by a third evaluator (SR). Each qualifying study was evaluated using the Joanna Briggs Institute Critical Appraisal Tools to assess methodological quality, considering the study type. This tool provides three levels of bias: unclear, low, and extreme. The risk is based on data explicitly, data partitioning, external validation, optimization technique, performance indicators, version details, and limitations.

### 4.1 Evaluation of the quality and risk of bias

Q1: Is the source of the dataset explicitly delineated?
Q2: Was the data corpus correctly allocated into training, validation, and test sets to avert data leakage?
Q3: Does the restricted use of a single public dataset without external validation on an independently sourced and separate dataset severely constrain the model’s shown generalizability and robustness to novel, unseen data?
Q4: How thoroughly are the selected optimization techniques articulated, and is there a coherent rationale for their selection and hyperparameters, model, and feature selection?
Q5: Do the performance indicators extend beyond mere accuracy to include precision, recall, *F*_1_-score, AUC, sensitivity, and specificity, thereby offering a thorough and impartial assessment of the model’s efficacy?
Q6: Is adequate information included regarding the DL architecture, particular libraries/frameworks, version numbers, and the training and optimization procedures to facilitate independent replication of the model’s development?
Q7: Does the study explicitly and critically address its limitations and potential biases?

### 4.2 Risk of bias scoring criteria and thresholds

The ultimate RoB assessment for each included study was classified according to the ratio of items designated as 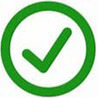, which means minimal RoB (higher quality): ≥70% of qualifying items were rated “low bias” or “moderate bias,” and the study obtained no more than one “unclear” rating in any specified crucial domain. High RoB (lower quality): less than 70% of qualifying items were rated 
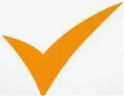
 ratings in two or more specified important domains. Extreme RoB: ≤50% of eligible items received a 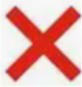 score, resulting in the exclusion of these studies from the data synthesis.

The risk of bias shown in Table 4 answers the aforesaid questions.

**Table 4:** Risk of bias.

Study ID	Question number							Overall, bias
	Q1	Q2	Q3	Q4	Q5	Q6	Q7	
Santos et al. (2022)	Unclear	Unclear	Extreme bias	Low bias	Low bias	Extreme bias	Low bias	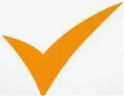
He et al. (2021)	Low bias	Low bias	Extreme bias	Low bias	Moderate bias	Low bias	Low bias	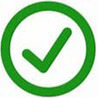
Zhang et al. (2022)	Low bias	Low bias	Extreme bias	Low bias	Low bias	Extreme bias	Low bias	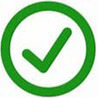
Gupta et al. (2022)	Low bias	Low bias	Extreme bias	Unclear	Low bias	Unclear	Unclear	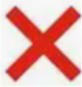
Khan et al. (2023)	Low bias	Low bias	Extreme bias	Low bias	Unclear	Extreme bias	Low bias	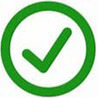
Khan et al. (2024)	Low bias	Low bias	Extreme bias	Low bias	Low bias	Low bias	Low bias	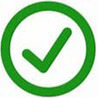
Purna Chandra Reddy and Gurrala (2022b)	Low bias	Unclear	Extreme bias	Low bias	Unclear	Extreme bias	Low bias	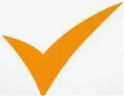
Wang et al. (2016)	Low bias	Low bias	Low bias	Moderate bias	Low bias	Moderate bias	Extreme bias	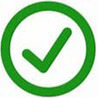
Purna Chandra Reddy and Gurrala (2022a)	Low bias	Low bias	Low bias	Low bias	Low bias	Low bias	Low bias	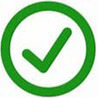
Purna Chandra Reddy and Gurrala (2023)	Low bias	Low bias	Extreme bias	Low bias	Low bias	Low bias	Low bias	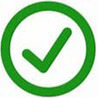
Reddy and Soma (2025)	Low bias	Low bias	Extreme bias	Low bias	Low bias	Low bias	Low bias	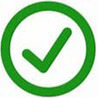
Naik et al. (2023)	Low bias	Low bias	Extreme bias	Moderate bias	Moderate bias	Unclear	Low bias	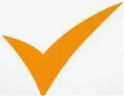
Karthik and Mahadevappa (2024)	Low bias	Low bias	Extreme bias	Low bias	Low bias	Low bias	Low bias	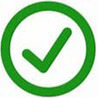
Mugada and Lakshmi (2025)	Low bias	Low bias	Extreme bias	Low bias	Low bias	Unclear	Low bias	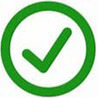
Navaneethan and Devarajan (2024a)	Low bias	Low bias	Low bias	Low bias	Low bias	Low bias	Low bias	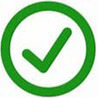
Ahmed et al. (2025)	Low bias	Low bias	Extreme bias	Low bias	Low bias	Moderate bias	Low bias	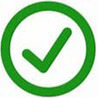
Rajagopalan et al. (2021)	Low bias	Moderate bias	Extreme bias	Low bias	Low bias	Low bias	Moderate bias	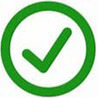
Krishna and Rao (2023)	Low bias	Low bias	Extreme bias	Low bias	Low bias	Low bias	Low bias	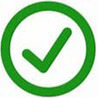
Reddy et al. (2023)	Unclear	Unclear	Extreme bias	Low bias	Unclear	Unclear	Low bias	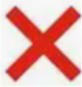
Aurangzeb et al. (2021)	Low bias	Unclear	Extreme bias	Low bias	Low bias	Unclear	Low bias	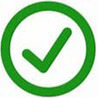
Jasper Gnana Chandran et al. (2023)	Low bias	Low bias	Extreme bias	Low bias	Low bias	Unclear	Unclear	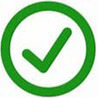
Sreejini and Govindan (2013)	Low bias	Unclear	Extreme bias	Low bias	Unclear	Extreme bias	Low bias	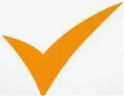
Elwin et al. (2022)	Low bias	Unclear	Extreme bias	Low bias	Unclear	Extreme bias	Low bias	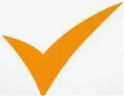
Lu et al. (2023)	Low bias	Unclear	Extreme bias	Low bias	Low bias	Unclear	Low bias	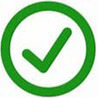
Devi and Karthikeyan (2024)	Low bias	Low bias	Extreme bias	Low bias	Low bias	Low bias	Low bias	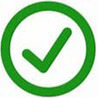
Zeng et al. (2021)	Low bias	Low bias	Extreme bias	Low bias	Low bias	Low bias	Low bias	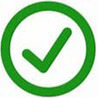
Rawat and Kumar (2024)	Low bias	Low bias	Extreme bias	Low bias	Low bias	Low bias	Low bias	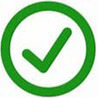
Chincholi and Koestler (2023)	Low bias	Low bias	Extreme bias	Low bias	Low bias	Low bias	Low bias	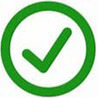
Bhimavarapu and Battineni (2022)	Low bias	Low bias	Extreme bias	Low bias	Low bias	Low bias	Low bias	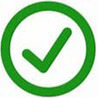

**Figure 9:**
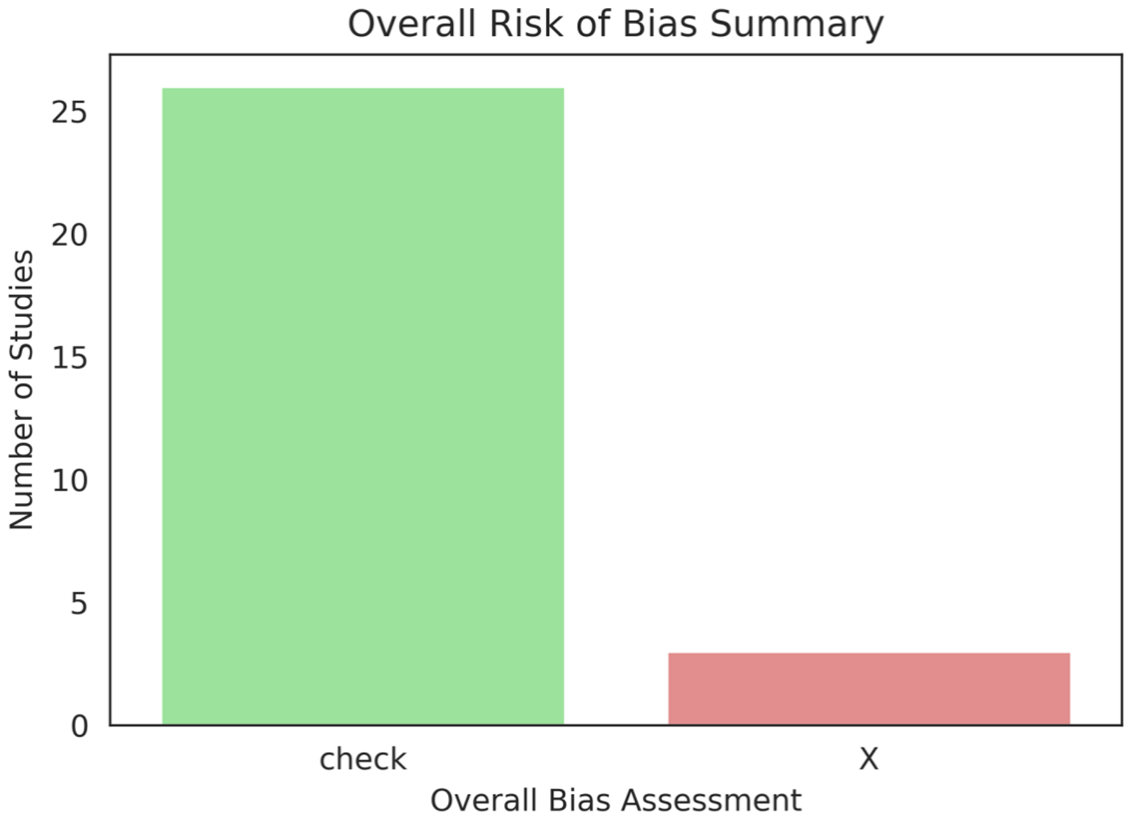
Risk bias summary.

Figure 9 visualizes the risk of bias.

The “Table: Risk of Bias” methodically assesses multiple studies based on seven fundamental questions. In Q1, concerning the specific identification of the dataset source, most research exhibits “Low Bias,” signifying transparent reporting, while a few are categorized as “Unclear.” Regarding the appropriate partitioning of datasets to prevent data leakage (Q2), most studies demonstrate “Low Bias,” indicating sound methodology; nonetheless, a significant number display “Extreme Bias” or are classified as “Unclear,” raising concerns about data integrity. Q3 highlights a significant limitation in generalizability stemming from the exclusive reliance on a singular public dataset without external validation, revealing a widespread concern, as numerous studies exhibit “Extreme Bias” and others “Moderate Bias,” underscoring a frequent deficiency in demonstrating robustness to new data. In Q4, regarding the comprehensive explanation of optimization strategies and their justifications, the responses are varied, featuring numerous “Low Bias” entries alongside notable occurrences of “Unclear” or “Extreme Bias,” signifying inconsistent reporting quality. Q5, emphasizing the extensive application of performance metrics beyond simple accuracy, generally indicates “Low Bias,” implying a robust commitment to comprehensive model evaluation in most investigations. Concerning Q6, the sufficiency of information for independent replication, akin to Q4, reveals a heterogeneous landscape, featuring both “Low Bias” and many “Unclear” or “Extreme Bias” entries. Ultimately, Q7, which evaluates whether the study explicitly and critically acknowledges its limitations and potential bias, reveals a troubling prevalence of “Extreme Bias” and “Unclear” ratings, signifying a pervasive inadequacy or ambiguity in recognizing study constraints. While certain methodological aspects, such as comprehensive performance metrics, are well-addressed. Ultimately, Q7, which evaluates whether the study explicitly and critically acknowledges its limitations and potential bias, reveals a troubling prevalence of “Extreme Bias” and “Unclear” ratings, signifying a pervasive inadequacy or ambiguity in recognizing study constraints. While certain methodological aspects, such as comprehensive performance metrics, are generally well-addressed, critical areas, including external validation for generalizability and transparent reporting of limitations and biases, often exhibit substantial concerns across the assessed studies, as indicated in the “Overall Bias” column, where studies are explicitly marked with an “X.”

### 4.3 Clinical implications

The advancement of lightweight CNN and other models designed for mobile and embedded devices demonstrates widespread applicability. This is essential for extensive screening in regions with constrained specialized healthcare services. Models that yield interpretable outputs, such as those showing identified lesions on fundus images (the YOLO-based method and the lightweight CNN), are crucial for aiding physicians and fostering trust and acceptance in clinical decision-making. Authentic high-performing models such as OHGCNet, the ACO-based OCT model, and the DeepID3 model were verified using actual clinical datasets, viz., IDRiD, Soonchunhyang University Hospital OCT data, which is essential for confirming their generalizability and dependability.

## 5 Discussion

The discussion section is based on the research questions framed for this study.

The assessed models use a variety of publicly accessible and clinical information, including fundus pictures and OCT. Fundus image analysis extensively uses recognized public datasets, including IDRiD, Messidor, APTOS-2019, EyePacs, and Dataset of Diabetic Retinopathy. For the diagnosis of DME, specialized OCT datasets, such as the Duke OCT Dataset, the extensive collection of images by Kermany et al. (2018), and clinical data from particular institutions (Qilu Hospital, Soonchunhyang University Bucheon Hospital) are applied by different authors. Although this diversity is beneficial, a persistent disadvantage is the reliance on a single dataset, frequently observed in individual studies using only DRIVE or IDRiD. The absence of multi-centric validation across diverse imaging protocols and patient demographics, such as the restricted size of the Qilu Hospital data or the 100-image Messidor subset, limits the generalizability of the models, highlighting the need for rigorous cross-dataset testing to ensure dependable implementation in various clinical environments. Research reports that the gap between validation and test performance demonstrates the model’s generalization challenges, particularly for small lesions like microaneurysms. The prevalence of models such as U-Net in medical picture segmentation is due to their architecture, which is specifically engineered for jobs necessitating accurate localization and context retention. The juxtaposition of meta-heuristic optimizers such as PSO and gradient-based optimizers like Adam indicates that each possesses distinct advantages, with PSO being notably proficient in hyperparameter tweaking and Adam demonstrating superiority in training DL models. Research findings suggest that the integration of sophisticated designs with efficient optimization methods might substantially enhance the identification and categorization of retinal disorders.

To bridge the gap between algorithmic innovation and clinical utility, some researchers have specifically enhanced diagnostic precision and diminished computational latency, which integrated within the operational framework of contemporary ophthalmic procedures. By reducing processing time, the model emerges as an effective instrument for high-throughput screening in primary care settings and telemedicine networks, where swift, real-time feedback is crucial for patient triage (Gupta et al., 2022). Practical application is underpinned by stringent system validation within hospital frameworks (Reddy and Soma, 2025), guaranteeing that the technology retains its efficacy when utilized by non-expert healthcare staff (Gupta et al., 2022). This efficient integration enables the early diagnosis of DME, transforming the clinical approach toward timely, vision-preserving therapies (Jasper Gnana Chandran et al., 2023). The use of explainability and interpretability qualities mitigates the “black box” characteristic of conventional AI, offering doctors a visible diagnostic reasoning essential for fostering physician trust and informed decision-making (Mugada and Lakshmi, 2025). To guarantee enduring scalability and address intrinsic difficulties like dataset variability and picture quality fluctuations across various devices, we employ versatile architectures capable of multi-modal integration (Rajagopalan et al., 2021). When integrated with established deployment methodologies (Naik et al., 2023), these technical advancements transition from theoretical enhancements to robust clinical solutions that eliminate conventional obstacles to AI adoption in diabetic eye care.

### 5.1 RQ1: Current trends and advancements

This research underscores the notable progress in the application of DL for medical imaging, particularly in OCT for retinal examination. The trend is shifting toward the integration of pixel-wise classification with structured surface extraction within a single framework, thereby improving the accuracy and efficiency of retinal layer segmentation. The suggested technique achieves sub-pixel precision and preserves topological integrity, which is essential for clinical applications. The integration of ML, ensemble learning, and optimization models is employed. The creation of CAD models to aid doctors in detecting AMD and DME, thereby alleviating workload and enhancing diagnostic precision, involves the integration of different models, the incorporation of self-attention layers, and the implementation of web-based applications for real-time illness prediction. Custom U-Net models facilitate segmentation, while data security is maintained through safe storage (Karthik et al., 2024). The Gaussian Adaptive Bilateral Filter and CLAHE are employed for image preprocessing to enhance quality by reducing noise while preserving edge integrity. Modified DeepLabV3+, a semantic segmentation model engineered to identify the macular region in OCT images in the reviewed articles. RegNet serves as a DL context for feature abstraction (Navaneethan et al., 2024b). Optimization of the DeepID3 network, utilization of pre-trained CNNs from ImageNet, and application of mathematical morphology for image preprocessing are used by Ahmed et al. (2025) in their work. Improvements in content-based image retrieval (CBIR) systems utilizing deep learning enhance diagnostic precision and efficiency by retrieving images based on content rather than metadata, as presented by Rajagopalan et al. (2021).

### 5.2 RQ2: Deep learning and machine learning models

The researchers use a fully convolutional network (FCN) as the principal model for segmenting retinal layers. This model is engineered to provide smooth, continuous surfaces with accurate anatomical sequencing. The architecture features a residual U-Net framework, adept at capturing spatial hierarchies within the data. Researchers also cite alternative models, such as random forests and graph-based methodologies, for comparative analysis. Linear regression (LR), support vector machine (SVM), K neighbors regressor, random forest regressor (RF), ridge regressor, and an ensemble model (LR + RF) (Naik et al., 2023) are employed, as well as Hybrid Neural Networks and Deep Convolutional Neural Networks (Karthik et al., 2024), Hybrid Graph Convolutional Networks (HGCN) (Pandugula et al., 2025), DenseNet-201, InceptionV3, and ResNet-50 (Navaneethan et al., 2024b) for model development. Lightweight models, attention mechanisms, patch-based implementations, and attention-based models are also utilized. Shape Index Histogram with Honey Badger Aquila Optimization (SIH + HBAO)-based deep convolutional networks, Adaptive Transfer Learning-based MobileNet (ATL-MobileNet), ShuffleNet V2, Dilated Mobile Transnet++ (Dil-M Transnet++), and Self-Attention Convolutional Neural Network (SACNN) are employed as classification models. The implementation of SVMs with sequential minimal optimization (SMO) for feature selection is also observed. The application of feature extraction methods, such as linear configuration patterns (LCP) and multi-scale feature extraction, improves classification efficacy. U-Net, InceptionV3, Xception, DenseNet201, ResNet50, VGG16, AlexNet, multi-scale CNN ensembles, deep residual networks, recurrent neural networks, Modified DeepLabV3+, and bespoke ensemble models are employed for the semantic segmentation of OCT images.

### 5.3 RQ3: Optimization algorithms

The research employs the Adam optimizer, Life Choice-Based Optimizer (LCBO), Grid Search, Stochastic Gradient Descent (SGD), Flower Pollination Optimization Algorithm (FPOA), and Arithmetic Optimization Algorithm (AOA) for training the DL model, utilizing specific hyperparameters, including an initial learning rate of 10^−4^ and weight decay of 10^−4^. Social Ski Operator Reference (Mugad et al., 2025) and the Random Variable Enhanced Humboldt Squid Optimization Algorithm (RVE-HSOA) are employed for weight optimization. Optimization algorithms, including Ant Colony Optimization (ACO), Modified Grey-Wolf Optimizer (MGWO), Convolutional Block Attention Modules (CBAM), Dingo Optimization Algorithm, and Joint Disease Attention (JDA), are utilized for feature selection. Filter optimization is employed in the convolution layer to mitigate overlapping and enhance convergence speed. The parameters of CLAHE are optimized using Modified Particle Swarm Optimization (MPSO) (Lu et al., 2023). The optimization process aims to improve the model’s efficacy in segmenting retinal layers and lesions, guaranteeing an end-to-end training approach, which represents a notable advancement over conventional methods necessitating post-processing. The utilization of the Capuchin Search Optimization Algorithm (CSOA) optimizes the parameters of the AGNN (Devi et al., 2024). The Autoregressive-Henry Gas Sailfish Optimization (Ar-HGSO) model exemplifies a shift toward the application of hybrid optimization methodologies to improve model efficacy (Zeng et al., 2021).

### 5.4 RQ4: Limitations

The principal constraints emphasized in the essay pertain to dataset challenges. Numerous research studies encounter data imbalance, restricted dataset size, and insufficient diversity. The reliance on a single dataset is frequently noted, which may result in bias and impede a model’s capacity to generalize to alternative populations or data sources. Additional constraints encompass computational difficulty, limited annotated datasets, and hardware restrictions. Certain approaches exhibit distinct vulnerabilities, such as the risk of overfitting, insufficient external validation, or dependence on image-level supervision instead of more comprehensive annotations.

### 5.5 RQ5: Performance metrics

The results reveal superior performance across multiple criteria for the DL models and optimization strategies examined. Accuracy metrics are regularly elevated, sometimes surpassing 90% and frequently attaining 98–99%. The sensitivity and specificity measures are generally high, often in the 90s, indicating effective identification of true positives and true negatives. *F*_1_-scores, which equilibrate precision and recall, are typically robust, frequently exceeding 90%; however, one instance exhibits a diminished *F*_1_-score of 0.2521. Certain outcomes additionally encompass specific metrics, such as mean absolute deviation (MAD) and root mean square error (RMSE) for particular tasks, alongside area under the curve (AUC) values, which are elevated to 0.996 and 0.8422 in specified circumstances. Moreover, there are references to diminished training epochs and decreased execution times, signifying enhancements in efficiency in certain instances.

### 5.6 RQ6: Research gaps

A notable research deficiency is the absence of generalizability in the models. This is chiefly due to an excessive dependence on a singular dataset in numerous investigations. A persistent requirement for external validation exists to confirm that the models function well on novel, unobserved data. Moreover, certain models are confined to particular tasks, such as being limited to 2D B-scans, and may have difficulties with specific visual intricacies, such as misunderstanding between the optic disc and exudates or the oversight of subtle lesions. The lack of varied datasets and comprehensive demographic data exacerbates the deficiency in model robustness and applicability.

### 5.7 Sankey analysis

**Figure 10:**
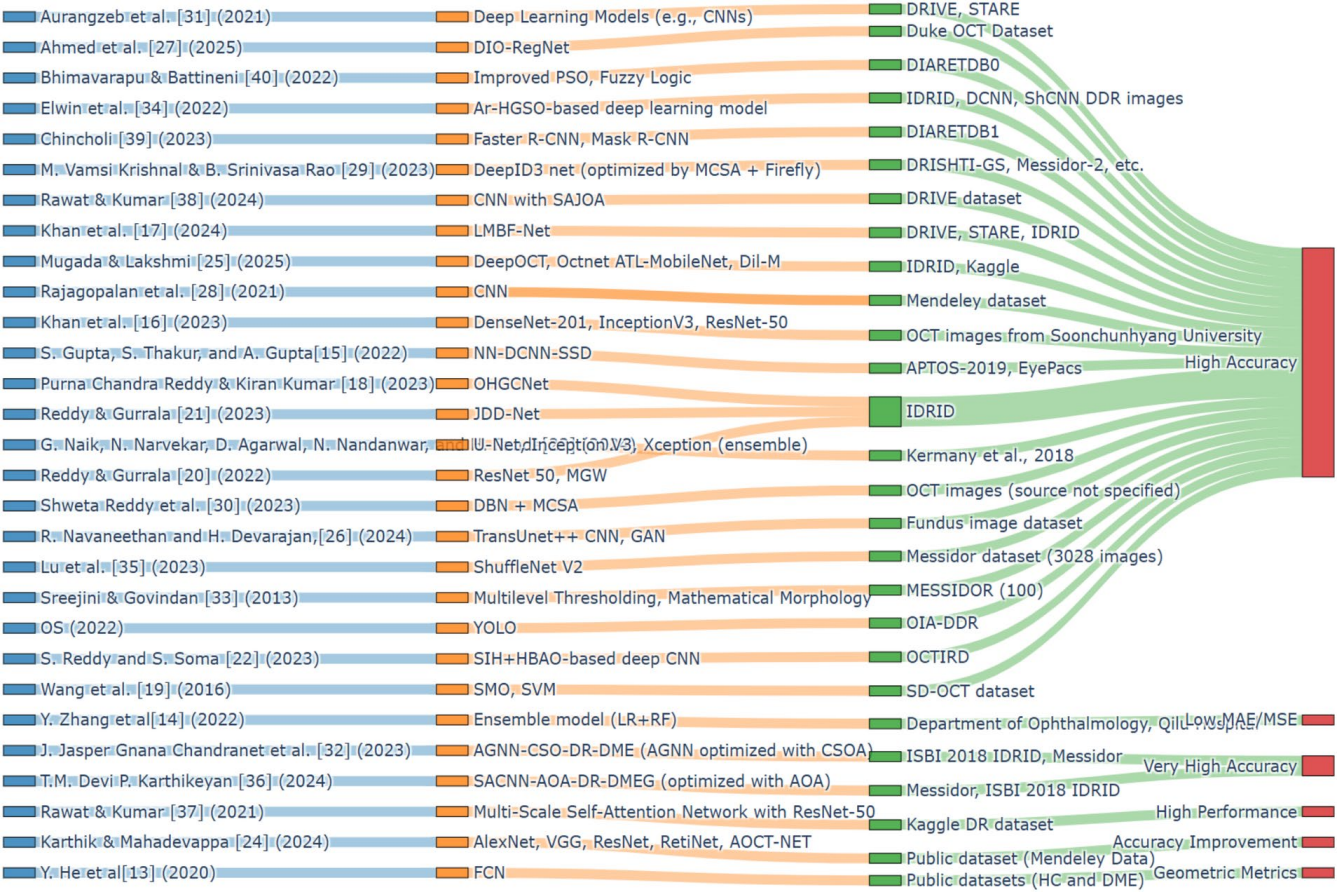
Sankey analysis.

A Sankey diagram (shown in Figure 10) visually depicts the flow of quantities among several entities, with the width of the connecting arrows corresponding to the size of the flow. This robust visualization tool successfully demonstrates the interrelated relationships in the research data. Authors, the models they employ, the datasets on which those models are applied, and the resultant outcomes are all indicated. By analyzing the differing widths of the links, one can readily identify the most prolific authors in particular domains, the models predominantly used with specific datasets, and the types of outcomes most frequently attained, thereby offering a quantitative summary of research contributions and trends across these essential components of scientific investigation.

### 5.8 Research challenges

To advance the domain of DME detection, a targeted “feature study” would meticulously analyze and tackle various interrelated challenges. Principal research avenues will focus on augmenting dataset quality and diversity, emphasizing the development of larger, more representative collections of retinal images that encompass diverse demographics and disease stages, while concurrently implementing stringent measures to standardize and enhance annotation consistency to mitigate bias and ensure reliable ground truth. Simultaneously, a primary emphasis will be placed on enhancing model generalizability and robustness through comprehensive external validation using independently sourced datasets, rather than depending solely on singular public collections. Additionally, the research will focus on enhancing the computational efficiency and scalability of algorithms by investigating sophisticated optimization methods and precise hyperparameter tuning to ensure practical clinical relevance and swift inference times. This study would promote and execute thorough performance evaluations using a wider array of metrics beyond mere accuracy. It would also investigate methods to enhance model interpretability and explainability, thereby building trust and enabling smooth integration into clinical workflows, to create highly effective and reliable diagnostic tools. The research challenges in DME detection mainly include data limitations, annotation quality, model generalizability, computational complexity, optimization techniques, performance evaluation, and the incorporation of advanced methodologies. Future research may aim to extend the model to include adjacent B-scan information, potentially improving the accuracy of surface segmentation in more intricate retinal geometries.

## 6 Conclusion

This systematic literature review comprehensively delineated the domain of optimization algorithms used for DME, employing a stringent methodology informed by Joanna Briggs tools and an exhaustive risk of bias evaluation, classifying studies as low, moderate, high, or no bias. Our analysis, visually supported by a network diagram illustrating keyword connections and a coherence diagram for optimization algorithms, uncovered a variety of methodologies encompassing model development, feature extraction, model selection, and feature selection, as demonstrated in our sunburst diagram of DL and ML models. We meticulously analyzed the preprocessing techniques implemented, the specific optimization methods applied for parameter tuning and weight adjustment, and the documented model performance metrics, establishing a clear connection between methodologies, authors, and results through our Sankey diagram. Importantly, although notable progress in automated DME management was recognized, a consistent limitation throughout the literature was the widespread dependence on singular, frequently restricted datasets, raising concerns about generalizability and the risk of labeling biases. Notwithstanding these challenges, the synthesized evidence emphasizes the transformative capacity of optimization algorithms in improving DME diagnosis, monitoring, and treatment, underscoring an urgent necessity for future research to concentrate on multi-center studies with varied, externally validated datasets to enable the integration of these promising algorithmic solutions into effective clinical practice. Based on our review, the domain is evolving from proof-of-concept research to clinically practical systems, with the most potential immediate applications being disease screening and severity assessment.

### 6.1 Future work

Future advancements should focus on enhancing microlesion detection using two-stage architectures with innovative preprocessing, developing explainable AI to facilitate clinical acceptance, and integrating multiple imaging modalities for comprehensive evaluation. The integration of conventional machine learning, DL, and metaheuristic optimization with transformer-based architectures and hybrid methodologies will demonstrate potential improved performance.

## Data Availability

The original contributions presented in the study are included in the article/supplementary material, further inquiries can be directed to the corresponding author.
